# Tetracycline, Sulfonamide, and Erythromycin Residues in Beef, Eggs, and Honey Sold as “Antibiotic-Free” Products in East Tennessee (USA) Farmers’ Markets

**DOI:** 10.3390/vetsci10040243

**Published:** 2023-03-24

**Authors:** Shamim Sarkar, Marcy J. Souza, Tomas Martin-Jimenez, Mohamed A. Abouelkhair, Stephen A. Kania, Chika C. Okafor

**Affiliations:** Department of Biomedical and Diagnostic Sciences, College of Veterinary Medicine, University of Tennessee, 2407 River Drive, Knoxville, TN 37996, USA

**Keywords:** antibiotic residues, tetracycline, sulfonamide, erythromycin, antimicrobial resistance, beef, egg, honey, ELISA, Farmers’ market, organic, antibiotic-free, East Tennessee

## Abstract

**Simple Summary:**

The increasing popularity of food products from farmers’ markets among consumers has been attributed to their belief that such food is more likely to be organic and free of harmful chemicals. The study’s objective was to measure antibiotic residues in “antibiotic-free” products sold at farmers’ markets in East Tennessee. We purchased “antibiotic-free” beef, egg, and honey products from farmers’ markets in East Tennessee and tested them for tetracycline, sulfonamide, and erythromycin residues. All tested food products contained varying levels of antibiotic residues. Median concentrations of these residues were below the maximum residue levels set in the U.S. for beef and eggs, indicating that these products are considered safe for consumption. But no residue limit has been set for honey products in the U.S. Because these “antibiotic-free” products should not have contained residues given their label, further research is necessary to determine the source of the residues in these foods.

**Abstract:**

Foods that contain antibiotic residues have potential adverse health effects on consumers and provide selective pressure for the threat of antimicrobial resistance (AMR). This study’s objective was to measure tetracycline, sulfonamide, and erythromycin residues in beef, eggs, and honey sold as “antibiotic-free” at farmers’ markets in East Tennessee (East TN) in the United States (U.S.). Between July and September 2020, 36 “antibiotic-free” food products (9 beef, 18 egg, and 9 honey products) were purchased from East TN farmers’ markets and tested for tetracycline, sulfonamide, and erythromycin residues using competitive enzyme-linked immunosorbent assays (cELISA). All beef, egg, and honey products had tetracycline residue; the median concentrations were 51.75, 30.25, and 77.86 µg/kg, respectively. Sulfonamide residue was present in every sample of beef. Of 18 eggs, 11 eggs had detectable sulfonamide residue; the median concentrations were 3.50 and 1.22 µg/kg in beef and eggs, respectively. Each sample of beef and honey contained erythromycin residue; the median concentrations were 3.67 and 0.68 µg/kg, respectively. Overall, the median concentrations of tetracycline, sulfonamide, and erythromycin residues were below the maximum residue levels (MRLs) set in the U.S. for beef and eggs. Thus, the beef and eggs sold as “antibiotic-free” in East TN farmers’ markets can be considered safe for consumption. Safety determination for honey could not be made because MRLs have not been set for honey in the U.S. Because these residues should not be expected in “antibiotic-free” food products, it is important to further investigate the potential sources of these residues in these products.

## 1. Introduction

Antibiotics are prescribed to treat, prevent, and control infectious illnesses in food-producing animals [1,2,3,4]. The global average yearly consumption of antibiotics per kilogram of animal produced is estimated at 45 mg/kg for cattle and 148 mg/kg for chickens [4,5]. The U.S. Food and Drug Administration (FDA) reports that approximately 6650 tons of medically important antibiotics were sold in the U.S. in 2018 for use in food-producing animals [6]. The most common antibiotics were tetracycline (66%, 4389 tons), penicillin (12%, 789 tons), macrolides (8%, 532 tons), aminoglycosides (5%, 332.5 tons), and sulfonamides (5%, 332.5 tons) in the U.S. [6]. Inappropriate use of antibiotics in food-producing animals is one factor associated with the presence of unsafe levels of antibiotic residues in foods of animal origin, such as meat, eggs, and honey [7]. There is evidence of unsafe levels of tetracycline and penicillin residues in Bob veal calves and culled cows in the U.S. [8]. However, to date, there are no available reports regarding the levels of antibiotic residues in foods of animal origin sold at farmers’ markets in the U.S. The presence of antibiotic residues in animal products over allowable limits is a potential driver of the development of antimicrobial-resistant (AMR) bacteria in humans [9,10,11,12], which is a growing global public health concern [13]. Annually in the U.S., the U.S. Centers for Disease Control and Prevention report more than 2.8 million AMR infections, resulting in more than 35,000 deaths in humans [14].

The U.S. FDA is concerned about the development of AMR in human and animal bacterial pathogens when medically important antibiotics are used in food-producing animals in an imprudent manner. Hence, the U.S. FDA updated the Veterinary Feed Directive (VFD) on 1 October 2015 and fully implemented it on 1 January 2017, in accordance with the FDA’s Guidance for Industry #213 [15], to ensure the judicious use of medically important antibiotics in food-producing animals in the U.S. [16]. A study reported that feed antibiotic sales decreased after the Veterinary Feed Directive (VFD) regulations were implemented in the U.S. [17]. However, according to Tennessee (TN) cattle producers, the VFD rule change could lead to the overuse of injectable antimicrobials in beef cattle, which would increase antibiotic residues [16]. These producers believe that violative antibiotic residues would occur more frequently in farmers’ markets compared to conventional grocery markets due to minimal regulatory oversight [16].

The number of farmers’ markets (a collection of two or more farm vendors selling agricultural products directly to customers at a common, recurrent physical location) [18] has increased steadily in the past decades in the U.S. [19,20]. In 2019, more than 3 million consumers shopped in about 9000 farmers’ markets in the U.S. [18,21], with Tennessee hosting over 40 farmers’ markets. The estimated sales of farmers’ markets products reached more than 1 billion USD in 2019 [22]. Growing consumer preference for food products from farmers’ markets has been linked to the consumer’s perception that foods purchased in these settings are more likely organic and free from destructive chemicals and pesticides [23,24,25], as well as being fresh, nutritious, and safe from pathogenic microbes [23,26,27,28,29] Countering these beliefs, Bellemare et al. found evidence of a positive correlation between the number of farmers’ markets and the rise in outbreaks of food-borne illness [30] in the U.S. Due to their popularity and the expectation of high-quality food products, the evidence of an association with increased food-borne illness and the suspicion that products could contain more antibiotic residues following the VFD rule change make food safety at farmers’ markets a paramount issue.

Since 1967, the U.S. national residue surveillance program for meat and poultry has collected samples and tested them for residues at slaughtering establishments to protect the health and welfare of consumers [31,32]. Results from this surveillance program from 1992–1996 indicated that the prevalence of tetracycline, sulfamethazine, and sulfadimethoxine residues were 5%, 8%, and 3%, respectively [32]. However, there are no antibiotic residue monitoring or surveillance programs or reports for eggs and honey sold at farmers’ markets intended for consumption in the U.S. Tetracycline, erythromycin, and sulfonamides are commonly used in food-producing animals and are among the medically important antibiotics for human health in the U.S. To our knowledge, the effect of the VFD rule change and the level of medically important antibiotic residues in foods of animal origin sold in farmers’ markets in the U.S has not been previously evaluated. Such exploration could validate how the VFD rule change has influenced responsible antibiotic use in animal agriculture.

With this background information, we aimed to answer the research question in this study: “Do antibiotic residues occur in beef, egg, and honey products sold at farmers’ markets as either “antibiotic-free” or “no-antibiotics” used during their production?” The “antibiotic-free” label refers to products derived from animals not given antibiotics at any stage of production, except in the case of illness with proper observation of a withdrawal period [33]. However, “no-antibiotics” means that animals were not given antibiotics during the production cycle of the product. For this report, all food products belonging to either of the categories described above were referred to as “antibiotic-free” [33]. This study’s objective was to quantify tetracycline, sulfonamide, and erythromycin residues in beef, eggs, and honey sold as “antibiotic-free” products at farmers’ markets in East Tennessee (East TN). Understanding the levels of antibiotic residues in these “antibiotic-free” food products sold at farmers’ markets is crucial in evaluating potential food safety concerns, thus contributing to consumer health protection.

## 2. Materials and Methods

### 2.1. Study Design, Area, and Sample Collection

**Figure 1:**
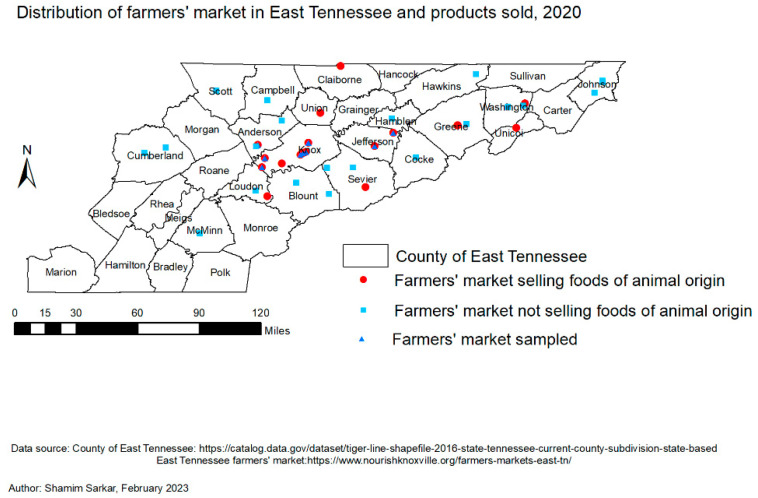
Map showing the distribution of farmers’ markets in East TN, those who do (n = 18) and do not sell (n = 23) foods of animal origin, and those from which purchased products (n = 9) were analyzed for antibiotic residues.

The required sample size for this study was determined based on the scheduled sampling plan outlined by the United States Department of Agriculture Food Safety and Inspection Service (USDA-FSIS) [34] for the target food products. Based on a 1% prevalence and 99% confidence level [34], the targeted sample size was 200 beef, 40 egg, and 40 honey products. Between July 2020 to September 2020 (the study period), there were 41 farmers’ markets spread across 22 counties in East TN, and 18 of these markets, located in 10 counties, offered foods of animal origin for consumer purchase. Vendors typically sold food products from farms located within about a 20-mile (32 km) radius of the farmers’ market. In the present study, farmers’ markets were purposively selected because they offered specific foods of animal origin of interest. Additionally, from these markets, those within a 100-mile (161 km) radius of the analysis laboratory were conveniently selected. Beef, eggs, and honey marketed as “antibiotic-free” products were purchased from vendors in the selected farmers’ markets. As indicated by the purchase receipts, no repeat food products were purchased from the same vendor throughout the study. Briefly, 9 beef, 18 egg, and 9 honey products were purchased from a total of 9 farmers’ markets within 3 counties of East TN (Figure 1). A unique identification (ID) was attached to each purchased product, indicating the market location, date, and food type. The products were maintained at the approved temperature for each product and transported to the laboratory within 3 h of purchase. Then, beef products were stored in a freezer at −80 °C, eggs were stored at refrigeration temperature [35], and honey products were kept at room temperature until analysis.

### 2.2. Sample Preparation for Detecting Tetracycline Residue

All beef, egg, and honey products were prepared and analyzed in duplicate, which allowed us to account for variation within the assay. The samples from the products were prepared according to the manufacturer’s instructions for the tetracycline competitive enzyme-linked immunosorbent assay (cELISA) kit (tetracycline ELISA Kit, E4273, Biovision, Milpitas, CA, USA) to quantitatively measure tetracycline residue in the foods. The cELISA kit provided the concentrations of tetracycline (0, 0.05, 0.15, 0.45, 1.35, and 4.05 µg/kg) used to construct the standard curve and all the reagents needed for the cELISA. The tetracycline cELISA has cross-reactivity with tetracyclines (100%), chlortetracycline (16.7%), oxytetracycline (10.7%), and doxycycline (4.2%). A brief description of beef, egg, and honey sample processing is as follows.

#### 2.2.1. Processing of Beef and Egg Products for the Tetracycline cELISA

First, 2 g of homogenized beef or egg product was placed into a 50 mL centrifuge tube. Next, 4 mL of 1% solution of trichloroacetic acid was added to the tube and the tube was oscillated for 2 min. Each sample was centrifuged at 4000 rpm at room temperature for 10 min. Finally, 1 mL of the supernatant solution was transferred to another tube for the subsequent cELISA.

#### 2.2.2. Processing of Honey Products for the Tetracycline cELISA

First, 1 g of each homogenized honey product was placed into a 50 mL centrifuge tube. Next, 2 mL of 1% solution of trichloroacetic acid was added to the tube, which was oscillated for 2 min. The sample was then centrifuged at 4000 rpm at room temperature for 10 min. Following centrifugation, 100 μL of supernatant was transferred to another centrifuge tube, then 1900 μL of the redissolving solution was added and mixed for 30 s. Finally, 1 mL of the supernatant solution was transferred to another tube for the subsequent cELISA.

### 2.3. Sample Preparation for Detecting Sulfonamide Residue

The sulfonamide residue cELISA kit (Sulfonamide ELISA kit, K4207, Biovision, California, USA) can detect sulfonamide residue in beef and egg products but is not validated for honey products. The cELISA kit provided sulfonamide concentrations of 0, 1, 3, 9, 27, and 81 µg/kg for generation of a standard curve and all the reagents needed for the subsequent cELISA. No cross-reactivity information was provided for the sulfonamide cELISA.

#### 2.3.1. Processing of Beef Products for the Sulfonamide cELISA

First, 1 g of each homogenized beef sample was added to a 50 mL centrifuge tube. Next, 5 mL of extraction solution was added to the tube, which was vortexed for 5 min. Then, the sample was centrifuged at 4000 rpm at room temperature for 10 min. Next, 200 μL of supernatant was transferred to another centrifuge tube, 200 μL of sample diluent was added, and the sample was mixed for 30 s. Finally, 50 μL of supernatant solution was transferred to another tube for the subsequent cELISA.

#### 2.3.2. Processing of Egg Products for the Sulfonamide cELISA

First, 1 g of homogenized egg sample (containing both yolk and white) was added to a 50 mL centrifuge tube. Next, 4 mL of distilled water was added to the tube, which was vortexed for 5 min. Afterward, 500 μL of the sample was mixed with 500 μL of extraction solution and mixed well. Finally, 50 μL of processed sample was taken in a tube for the subsequent cELISA.

### 2.4. Sample Preparation for Detecting Erythromycin Residue

The erythromycin cELISA kit (erythromycin ELISA kit, DEIABL-QB20, CD creative diagnostics, New York, NY, USA) can detect erythromycin residue in beef and honey products but is not validated for egg products. The cELISA kit provided standard concentrations of erythromycin (0, 0.2, 0.6, 1.8, 5.4, 16.2 µg/kg) for generation of a standard curve and all the reagents needed for the subsequent cELISA. Erythromycin cELISA has cross-reactivity with erythromycin (100%), erythromycin sulphate (114%) tylosin (<0.1%), timicosin (<0.1%), and spiramycin (<0.1%).

#### 2.4.1. Processing of Beef Products for the Erythromycin cELISA

First, 2 g of homogenized beef product was added to a 50 mL centrifuge tube. Next, 10 mL of acetonitrile–0.1 M NaOH solution was added, and the sample was vortexed for 10 min. Then, the sample was centrifuged at 3000× *g* at ambient temperature for 5 min. Following centrifugation, 1 mL of supernatant was transferred to a 15 mL centrifuge tube, 1 mL of n-hexane was added, the mixture was vortexed for 30 s, then 1 mL of extraction solution was added. Afterwards, the sample was centrifuged at 3000× *g* at ambient temperature for 5 min. Lastly, 1 mL of the lower layer of solution was transferred to a 2 mL tube for the assay.

#### 2.4.2. Processing of Honey Products for the Erythromycin cELISA

First, 2 g of honey product was added to a 50 mL centrifuge tube. Next, 2 mL of 0.1 M CB solution was added to completely dissolve the sample. Then, 6 mL ethyl acetate was added to the tube and mixed for 5 min. Then, the sample was centrifuged at 3000× *g* at ambient temperature for 5 min. Following centrifugation, 3 mL of the supernatant solution was transferred to the 15 mL centrifuge tube, 0.5 mL extraction solution was added, then the mixture was vortexed for 5 min to completely dissolve the sample. Lastly, 1 mL of each processed sample was taken in a 2 mL tube for the subsequent cELISA.

### 2.5. Competitive ELISA Steps

The cELISA assay for tetracycline was carried out according to the manufacturer’s instructions to determine the levels of tetracycline residue (Tetracycline ELISA Kit, Biovision, CA, USA). In brief, 50 μL prepared sample was added to each well (precoated with antibody against analyte of interest) of an ELISA plate. Then, 50 μL of antibody working solution was added to the well. Next, the plate was oscillated for 5 s, covered, and incubated in the dark for 30 min at 37 °C. After the incubation, the solution from the plate was discarded, and the plate was washed five times with 1X wash solution. Subsequently, 100 μL of enzyme conjugate was added to each well precoated with tetracycline antibody and incubated for 30 min at 37 °C. The plate was rewashed, 50 μL of Substrate A and 50 μL of Substrate B solution were added to each well, then the plate was oscillated gently for 5 sec. The plate was then incubated for 15 min at 37 °C. After the incubation, 50 μL stop solution was added to each well and oscillated gently to stop the reaction. Finally, the optical density of the sample was measured at 450 nm by a spectrophotometer within 10 min.

Similarly, the cELISAs for sulfonamide and erythromycin residues were carried out according to the manufacturer’s instructions. All standard concentrations of antibiotics that accompanied the respective kits were used for the generation of standard curves. Antibiotic concentrations were determined from the standard curves using non-linear curve fitting with Sigmoidal 4PL using GraphPad Prism version 9.5.1 (La Jolla, San Diego, CA, USA).

### 2.6. Data Analysis

Tetracycline, sulfonamide, and erythromycin residues were the target antibiotics for this study, and their residue concentrations were the three distinct continuous outcome variables for the study. Each continuous outcome variable was assessed for normality of distribution using the Shapiro–Wilk test. Medians and interquartile ranges (IQR) were calculated for non-normal distributions of continuous outcome variables. To examine the difference between food groups and the outcome variables, assumptions of normality and homogeneity of variance of tetracycline, sulfonamide, and erythromycin residues were assessed using the Shapiro–Wilk and Levene’s tests, respectively. Other than erythromycin residue, the other residues did not meet the assumption of normality in their residue concentrations. As a result, the statistically significant variations in the median concentrations of tetracycline residue among the three food groups (beef, eggs, and honey) were compared using the Kruskal–Wallis test [36]. Since the Kruskal–Wallis test revealed a significant difference, pairwise comparisons were next subjected to post hoc tests using Dunn’s test with a Bonferroni correction (to adjust the *p*-values that resulted from the multiple comparisons) [37]. The concentrations of sulfonamide (found in beef and egg products) and erythromycin (found in beef and honey products) were compared between the two compared food groups using the Mann–Whitney test. The concentrations of erythromycin (found in beef and honey products) were compared between the two food groups using the two-sample *t*-test. The statistical significance was assessed using a critical *p*-value of *p* < 0.05. The statistical analyses were performed in Stata 16.1 [38] and GraphPad Prism 9.5.1 software.

## 3. Results

**Figure 2:**
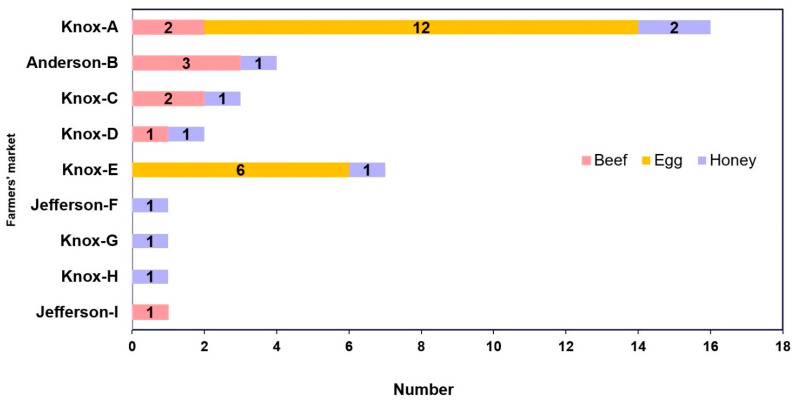
Distribution of food samples by farmers’ market (n = 36) from July to September 2020 in Eastern Tennessee (USA).

Of the nine farmers’ markets with food products tested for tetracycline, erythromycin, and sulfonamide antibiotic residues, most came from Knox County (Figure 1 and Figure 2) during the summer months of 2020. A total of 36 food products were purchased from 21 different vendors that marketed their food items as “antibiotic-free” during the study.

### Level of Antibiotics Residue in the Foods

All beef, egg, and honey samples had tetracycline residue. The median concentrations of tetracycline residue were 51.75, 30.25, and 77.86 µg/kg in beef, egg, and honey products, respectively (Table 1). None of the beef and egg products exceeded the MRL for tetracycline set by the U.S. FDA in this study (Table 1). The Kruskal–Wallis test results showed a statistically significant difference in the median tetracycline residue level between beef, eggs, and honey (*p* = 0.0315) (Table 1). Pairwise comparisons using Dunn’s test yielded results indicating a statistically significant difference between concentrations of tetracycline residue in honey and eggs (*p* = 0.0395). Likewise, Dunn’s test results suggested that the concentration of tetracycline residue was borderline higher in beef than in eggs (*p* = 0.0582).

Likewise, all beef products contained sulfonamide residue. Of 18 eggs tests, 11 eggs contained detectable sulfonamide residue. The median concentrations of sulfonamide residue were 3.50 and 1.22 µg/kg in beef and eggs, respectively. None of the beef and egg samples exceeded the MRL for sulfonamide set by the U.S. FDA (Table 1). The Mann–Whitney test results showed a statistically significant difference between the median sulfonamide residue level in beef and eggs (*p* = 0.0260) (Table 1).

Erythromycin residue was present in every sample of beef and honey; the median concentrations were 3.67 and 0.68 µg/kg, respectively. None of the beef samples exceeded the MRL for erythromycin set by the U.S. FDA (Table 1). The two-sample *t*-test test results showed that the mean erythromycin residue level was statistically higher in beef than in honey (*p* = 0.0004).

## 4. Discussion

To the authors’ best knowledge, this study provides the first measurement of tetracycline, sulfonamide, and erythromycin residues in beef, eggs, and honey sold at farmers’ markets for human consumption in East TN. The study findings revealed the presence of tetracycline, sulfonamide, and erythromycin residues with varied concentrations in beef, eggs, and honey sold as “antibiotic-free” products at farmers’ markets in East TN. Although the levels of the residues were lower than the MRLs set by the U.S. FDA in beef and eggs, these foods should be free of any antibiotic residue because they should have been produced without any antibiotics. There is no antibiotic residue monitoring or surveillance program for eggs obtained from backyard poultry and these products are readily sold at farmers’ markets in the U.S.

The presence of tetracycline, sulfonamide, and erythromycin residues in foods sold as “antibiotic-free” products at farmers’ markets can be linked to direct or indirect antibiotic exposure in food-producing animals. Direct exposure can occur in foods when food-producing animals are treated with antibiotics and a withdrawal period is not observed. This observation in the present study was also reported in another recent study in which beef “raised without antibiotics” tested positive for tetracycline residue in USDA-inspected slaughterhouses in the U.S. [39]. Backyard chickens can be exposed to antibiotics when they are directly injected with antibiotics or antibiotics are supplemented through their diets or water, resulting in antibiotic residues in the eggs. According to an experimental study, tetracycline residue in eggs has been linked to a tetracycline-supplemented diet [40]. Tetracycline is one of the most used, medically important antibiotics in food-producing animals [41], including honey production, in the U.S. [42]. There is evidence that tetracycline and erythromycin have been used against bacterial diseases such as American foulbrood (AFB) in honeybees [42]. However, because the food products came from “antibiotic-free” food-producing animals, direct exposure may be an unlikely cause in those circumstances, unless there is lack of integrity of “antibiotic-free” claims at farmers’ markets in the study areas.

Therefore, indirect exposure appears to be the most plausible source of the residues observed in the present study. Indirect antibiotic exposure can occur in foods when food-producing animals are exposed to antibiotics through environmental contamination and other agricultural farming activities. There is evidence showing environmental antibiotic contaminants can be associated with the presence of intensive livestock production, their sewage discharge [43,44], and other agricultural farming activities close to honey production [45,46,47]. For instance, when commercial farmers use antibiotics adjacent to antibiotic-free farms, water sources, soil, or inputs used on the antibiotic-free farms could be contaminated [48]. Hence, beef cattle may ingest antibiotic-contaminated water or animal feed, thereby increasing the likelihood of antibiotic residue in the beef. Similarly, indirect antibiotic exposure can occur in backyard chickens [35,49] when the chickens inadvertently consume antibiotic-containing ingredients. For instance, backyard chickens can eat excrement from cattle that has recently received oral antibiotic treatment, resulting in antibiotic residues in the eggs. Furthermore, honey can be exposed to antibiotic residues during honeybee foraging activities or contaminated through food sources such as nectar or pollen [50]. Additionally, honeybees can be exposed to water contaminated with erythromycin, tetracycline, or both types of antibiotics [43,51], resulting in antibiotic residues in the honey.

We were unable to find any published reports regarding levels of tetracycline and erythromycin residues in honey sold as “antibiotic-free” or “organic” products in farmers’ markets in the U.S. Several countries reported antibiotic residues in honey in Europe [47,52,53], Asia [54,55,56,57,58,59,60], and other countries [61,62]. Our study results can serve as baseline information that can help fill in the knowledge gap and guide future research to identify the exposure source and pathways of tetracycline and erythromycin residues in honey, including beekeepers’ knowledge level regarding antibiotic residues. The regulations and restrictions on antibiotic use in honeybees can vary widely by region and country. Currently, no MRLs are defined for erythromycin and tetracycline in honey products by the U.S. FDA [42,63,64] whereas the use of antibiotics in honeybees is either restricted or prohibited in various regions and countries around the world. For example, tetracycline and other antibiotics are not authorized for treatment of honeybees in the European Union [65]. Because the safety implications of the residues in our honey products could not be explained, it is recommended that MRLs for tetracycline and erythromycin in honey be established in the U.S. This would allow for the meaningful interpretation of the present results, clarifying what concentrations are safe in honey for consumers in the U.S.

However, there is a wide-ranging body of literature on tetracycline and sulfonamide residues in beef and eggs sold in conventional retail markets using different sampling and testing methods. Hence, this study’s findings cannot compare or contrast with international studies. The tetracycline and sulfonamide residue levels were below the MRLs set by the U.S. FDA in beef and eggs [66]; however, the beef and eggs in the present study should be free from tetracycline and sulfonamide residues because of the product labels. Likewise, all the beef samples were found to contain erythromycin residue, but the residue levels were below the MRL set by the U.S. FDA in beef [66]. These results indicated that beef and eggs sold at farmers’ markets were safe for consumers, considering the tetracycline, erythromycin, and sulfonamide residues identified during the study period, but repeated and prolonged exposure may be a health worry.

The low levels of tetracycline, sulfonamide, and erythromycin residues in foods of animal origin may pose an adverse health risk to consumers. For example, consumers exposed to low levels of antibiotic residues via foods of animal origin may provide selective pressure for AMR in bacteria in humans [67,68,69]. AMR bacterial infections can increase human medical costs, lengthen hospital stays, and increase the number of deaths. Furthermore, the U.S. FDA has reported four potential adverse health effects caused by antibiotic residues in foods of animal origin: changes in the metabolic activity of the intestinal microflora, changes in the number and composition of intestinal microorganisms, changes in the microflora’s AMR patterns, and changes in the microflora’s barrier effect [70]. Moreover, a study reported that the obesity epidemic in the U.S. could be due to the constant exposure of Americans to foods containing low-residue antimicrobial agents by disrupting the equilibrium state of the intestinal microbiota [71]. Future research should investigate the levels of tetracycline, sulfonamide, and erythromycin residues in beef, eggs, and honey sold in conventional stores without the “antibiotic-free” claim/label. The results of that study would help in clarifying whether the antibiotic residues in the present study were obtained potentially through direct or indirect exposure.

cELISA was used to quantify the antibiotic residue concentrations in the food samples because it is a relatively simple, rapid, and low-cost test compared to high-performance liquid chromatography (HPLC), liquid chromatography–mass spectrometry (LC-MS), or mass spectrometry (MS). In addition to being the most frequently used method, cELISA is easily operated on many food samples in a laboratory [72]. However, cELISA may not be as sensitive as other methods (HPLC, LC-MS, or MS) in the estimation of residues in food products. Prior studies showed that the recovery (%) of antibiotic residues in food matrices varied based on the sample and laboratory testing method [73], and most published literature reported the recovery (%) of different antibiotics in food matrices based on the HPLC method. A method such as HPLC was not used for further confirmation of cELISA-positive food samples due to budgetary and logistical constraints. Because a strong significant correlation (r-value of 0.93 at *p* < 0.01) between the HPLC and cELISA methods in measuring the concentration of tetracycline residue in foods of animal origin has been reported [72], it can be assumed that our results are comparable to those validated with HPLC.

There were some limitations associated with this study’s sampling. First, we conducted a cross-sectional study utilizing non-probability sampling (both purposive and convenience sampling) approaches to collect food products instead of a probability sampling method. Although the purposive sampling approach of selecting only farmers’ markets selling foods of animal origin of interest may have helped us to quickly identify our target food products, the convenience sampling approach of purchasing from these markets within a 100-mile (161 km) radius of the analysis laboratory may have introduced selection bias, thus affecting the generalization of our study results. However, the distribution in Figure 1 does not suggest that any such bias was significant. Moreover, we sampled from 50% of farmers’ markets selling foods of animal origin, which could help the generalization of our results. Second, vendors usually sold food products that were obtained from farms located within a radius of approximately 20 miles (32 km) from the farmers’ markets. While it was possible for food vendors to sell their food products at different farmers’ markets, based on the purchase receipts in this study, no food products were purchased more than once from the same vendor. Hence, the possible bias that would arise due to repeat products being purchased from the same vendor was not observed in the present study. Finally, the a priori required number of food product was not achieved because the food product collection period occurred during the incline phase of the COVID-19 pandemic. Notably, beef, egg, and honey supplies in farmers’ markets in the U.S. were severely impacted by the COVID-19 pandemic [74]. Since there were not enough foods available in the markets throughout the study period, we were unable to collect the necessary number of beef, egg, and honey products, negating the use of a probability sampling method. However, the 36 food products we collected appeared to be a good representation of such products at farmers’ markets in East TN.

## 5. Conclusions

The study results showed that the median concentrations of tetracycline, sulfonamide, and erythromycin residues were below the MRLs in beef and eggs sold as “antibiotic-free” products at farmers’ markets in East TN. Hence, these beef and eggs products can be considered safe for consumption. Since there are no established MRLs for erythromycin and tetracycline in honey in the U.S., we could not conclude whether the residues found in honey were considered safe or not. Although the antibiotic residues in the beef, eggs, and honey products did not exceed the MRLs, the residues should not have been present in the food products because they were sold as “antibiotic-free” products. More importantly, setting MRLs for erythromycin and tetracycline in honey is recommended to help us understand what concentrations of these residues in honey products are safe for consumers in the U.S. The study findings contribute to filling the knowledge gap about the levels of antimicrobial residues in beef, eggs, and honey sold as “antibiotic-free” products at farmers’ markets in East TN. Investigating the sources of the aforementioned residues in the targeted food products is paramount for future studies.

## Figures and Tables

**Table 1:** Tetracycline, sulfonamide, and erythromycin residues in foods of animal origin sold in farmers’ markets in East Tennessee (USA) during summer 2020.

Type of Sample	Total Samples	x/n	Tetracycline				
			Concentrations (µg/kg)	MRL ^¥^ (µg/kg)	MRL ^€^ (µg/kg)	Exceed MRL (U.S. FDA) n (%)	*p*-Value
			Median (IQR)				
Beef	9	9/9	51.75 (48.34, 56.61)	200	100	0 (0)	0.0315 ^a^
Egg	18	18/18	30.25 (12.22, 51.63)	200	200	0 (0)	
Honey	9	9/9	77.86 (23.34, 161.05)	NA	NA	NA	
	**Sulfonamide**						
Beef	9	9/9	3.50 (3.08, 4.40)	100	100		
Egg	18	11/18	1.22 (0.00, 3.55)	100	NA		0.0260 ^b^
	**Erythromycin**						
Beef	9	9/9	3.67 (3.41, 5.88)	100	200	0 (0)	
Honey	9	9/9	0.68 (0.40, 2.54)	NA	NA	NA	0.0004 ^c^

MRL = Maximum residue level. NA = Not applicable. IQR = interquartile range. x, Number of samples had antibiotic residues. n, Number of samples tested. ^a^ *p* values were obtained from the Kruskal–Wallis test. ^b^ *p*-values were obtained from the Mann–Whitney test. ^c^ *p*-values were obtained from the two-sample *t*-test. ^¥^ MRL set by the U.S. FDA. ^€^ MRL set by the European Union. For sulfonamide, “0” means below the limit of detection.

## Data Availability

The data are presented in this study are available on request from corresponding author.

## References

[1] Bacanlı M., Başaran N. (2019). Importance of antibiotic residues in animal food. Food Chem. Toxicol..

[2] Cordle M.K. (1988). USDA Regulation of Residues in Meat and Poultry Products. J. Anim. Sci..

[3] Ma F., Xu S., Tang Z., Li Z., Zhang L. (2021). Use of antimicrobials in food animals and impact of transmission of antimicrobial resistance on humans. Biosaf. Health.

[4] Tiseo K., Huber L., Gilbert M., Robinson T., Van Boeckel T. (2020). Global Trends in Antimicrobial Use in Food Animals from 2017 to 2030. Antibiotics.

[5] Van Boeckel T.P., Brower C., Gilbert M., Grenfell B.T., Levin S.A., Robinson T.P., Teillant A., Laxminarayan R. (2015). Global trends in antimicrobial use in food animals. Proc. Natl. Acad. Sci. USA.

[6] U.S. Food and Drug Administration (FDA). Center for Veterinary Medicine. 2018 Summary Report on Antimicrobials Sold or Distributed for Use in Food-Producing Animals. https://www.fda.gov/media/133411/download.

[7] Manyi-Loh C., Mamphweli S., Meyer E., Okoh A. (2018). Antibiotic use in agriculture and its consequential resistance in environmental sources: Potential public health implications. Molecules.

[8] Gibbons S.N., Kaneene J.B., Lloyd J.W. (1996). Patterns of chemical residues detected in US beef carcasses between 1991 and 1993. J. Am. Vet. Med. Assoc..

[9] Holmes A.H., Moore L.S.P., Sundsfjord A., Steinbakk M., Regmi S., Karkey A., Guerin P.J., Piddock L.J.V. (2016). Understanding the mechanisms and drivers of antimicrobial resistance. Lancet.

[10] Lee M.H., Lee H.J., Ryu P.D. (2001). Public Health Risks: Chemical and Antibiotic Residues—Review. Asian-Australas. J. Anim. Sci..

[11] Muaz K., Riaz M., Akhtar S., Park S., Ismail A. (2018). Antibiotic Residues in Chicken Meat: Global Prevalence, Threats, and Decontamination Strategies: A Review. J. Food Prot..

[12] Menkem Z.E., Ngangom B.L., Tamunjoh S.S.A., Boyom F.F. (2018). Antibiotic residues in food animals: Public health concern. Acta Ecol. Sin..

[13] Ferri M., Ranucci E., Romagnoli P., Giaccone V. (2015). Antimicrobial resistance: A global emerging threat to public health systems. Crit. Rev. Food Sci. Nutr..

[14] U.S. Centers for Disease Control and Prevention, National Center for Emerging and Zoonotic Infectious Diseases (NCEZID), Division of Healthcare Quality Promotion (DHQP). about Antimicrobial Resistance. https://www.cdc.gov/drugresistance/about.html.

[15] FDA, U.S. Guidance for Industry# 213, New Animal Drugs and New Animal Drug Combination Products Administered in or on Medicated Feed or Drinking Water of Food-Producing Animals: Recommendations for Drug Sponsors for Voluntarily Aligning Product Use Conditions with GFI# 209. Center for Veterinary Medicine: Rockville, MD, USA. https://www.fda.gov/media/83488/download.

[16] Ekakoro J.E., Caldwell M., Strand E.B., Okafor C.C. (2019). Perceptions of Tennessee cattle producers regarding the Veterinary Feed Directive. PLoS ONE.

[17] Dillon M.E. (2020). The Impact of Restricting Antibiotic Use in Livestock: Using a ‘One Health’ Approach to Analyze Effects of the Veterinary Feed Directive. Master’s Thesis.

[18] National Farmers Market Managers 2019 Summary (August 2020). USDA, National Agricultural Statistics Service. https://usda.library.cornell.edu/concern/publications/pz50hd694?locale=en.

[19] Eastwood D.B., Brooker J.R., Gray M.D. (1999). Location and other market attributes affecting farmer’s market patronage: The case of Tennessee. J. Food Distrib. Res..

[20] Phillips E. (2019). Chapter Ten. The Growing Trend of Farmers’ Markets in the United States (6–10). Case Studies in Food Policy for Developing Countries.

[21] USDA (2019). National Count of Farmers Market Directory Listings.

[22] USDA. Economic Research Service using data from USDA, Agricultural Marketing Service, National Agricultural Statistics Service, Farmers Markets and National Farmers Market Directory. https://www.ers.usda.gov/data-products/charts-of-note/charts-of-note/?topicId=f5a7d42d-5209-47db-abbb-2e2cc3634cde.

[23] Byker C., Shanks C.B., Misyak S., Serrano E. (2012). Characterizing Farmers’ Market Shoppers: A Literature Review. J. Hunger. Environ. Nutr..

[24] Conner D.S., Montri A.D., Montri D.N., Hamm M.W. (2009). Consumer demand for local produce at extended season farmers’ markets: Guiding farmer marketing strategies. Renew. Agric. Food Syst..

[25] Velasquez C., Eastman C., Masiunas J. (2005). An assessment of Illinois farmers’ market patrons’ perceptions of locally-grown vegetables. J. Veg. Sci..

[26] Feldmann C., Hamm U. (2015). Consumers’ perceptions and preferences for local food: A review. Food Qual. Prefer..

[27] Harvey R.R., Zakhour C.M., Gould L.H. (2016). Foodborne Disease Outbreaks Associated with Organic Foods in the United States. J. Food Prot..

[28] Wolf M.M., Spittler A., Ahern J. (2005). A profile of farmers’ market consumers and the perceived advantages of produce sold at farmers’ markets. J. Food Distrib. Res..

[29] Yu H., Gibson K.E., Wright K.G., Neal J.A., Sirsat S.A. (2017). Food safety and food quality perceptions of farmers’ market consumers in the United States. Food Control.

[30] Bellemare M.F., Nguyen N. (2018). Farmers markets and food-borne illness. Am. J. Agric. Econ..

[31] (2019). U.S. National Residue Program. https://www.fsis.usda.gov/node/1982.

[32] Paige J.C., Chaudry M.H., Pell F.M. (1999). Federal surveillance of veterinary drugs and chemical residues (with recent data). Vet. Clin. N. Am. Food Anim. Pract..

[33] Food Safety and Inspection Service Labeling Guideline on Documentation Needed to Substantiate Animal Raising Claims for Label Submissions. December 2019. https://www.fsis.usda.gov/sites/default/files/media_file/2021-02/RaisingClaims.pdf.

[34] USDA FSIS Office of Public Health Science, United States National Residue Program for Meat, Poultry, and Egg Products, 2019 Residue Sampling Plans. 1 October 1 2018 to 30 September 2019. https://www.fsis.usda.gov/sites/default/files/media_file/2020-07/fy2019-red-book.pdf.

[35] Cornejo J., Pokrant E., Figueroa F., Riquelme R., Galdames P., Di Pillo F., Jimenez-Bluhm P., Hamilton-West C. (2020). Assessing Antibiotic Residues in Poultry Eggs from Backyard Production Systems in Chile, First Approach to a Non-Addressed Issue in Farm Animals. Animals.

[36] Cleophas T.J., Zwinderman A.H. (2016). Non-parametric tests for Three or more samples (friedman and kruskal-Wallis). Clinical Data Analysis on a Pocket Calculator.

[37] Hochberg Y. (1988). A sharper Bonferroni procedure for multiple tests of significance. Biometrika.

[38] StataCorp (2019). Stata Statistical Software: Release 16.

[39] Price L.B., Rogers L., Lo K. (2022). Policy reforms for antibiotic use claims in livestock. Science.

[40] Dipeolu M.A., Eruvbetine D., Oguntona E.B., Bankole O.O., Sowunmi K.S. (2005). Comparison of effects of antibiotics and enzyme inclusion in diets of laying birds. Arch. Zootec..

[41] Patel S.J., Wellington M., Shah R.M., Ferreira M.J. (2020). Antibiotic Stewardship in Food-producing Animals: Challenges, Progress, and Opportunities. Clin. Ther..

[42] Reybroeck W., Daeseleire E., De Brabander H.F., Herman L. (2012). Antimicrobials in beekeeping. Vet. Microbiol..

[43] Karthikeyan K., Meyer M.T. (2006). Occurrence of antibiotics in wastewater treatment facilities in Wisconsin, USA. Sci. Total. Environ..

[44] Kimosop S.J., Getenga Z.M., Orata F., Okello V.A., Cheruiyot J.K. (2016). Residue levels and discharge loads of antibiotics in wastewater treatment plants (WWTPs), hospital lagoons, and rivers within Lake Victoria Basin, Kenya. Environ. Monit. Assess..

[45] Bonerba E., Panseri S., Arioli F., Nobile M., Terio V., Di Cesare F., Tantillo G., Chiesa L.M. (2021). Determination of antibiotic residues in honey in relation to different potential sources and relevance for food inspection. Food Chem..

[46] Rothrock M.J., Min B.R., Castleberry L., Waldrip H., Parker D., Brauer D., Pitta D., Nagaraju I. (2021). Antibiotic resistance, antimicrobial residues and bacterial community diversity in pasture-raised poultry, swine and beef cattle manures. J. Anim. Sci..

[47] Savarino A., Terio V., Barrasso R., Ceci E., Panseri S., Chiesa L.M., Bonerba E. (2020). Occurrence of antibiotic residues in Apulian honey: Potential risk of environmental pollution by antibiotics. Ital. J. Food Saf..

[48] Hanna N., Sun P., Sun Q., Li X., Yang X., Ji X., Zou H., Ottoson J., Nilsson L.E., Berglund B. (2018). Presence of antibiotic residues in various environmental compartments of Shandong province in eastern China: Its potential for resistance development and ecological and human risk. Environ. Int..

[49] Braykov N.P., Eisenberg J.N.S., Grossman M., Zhang L., Vasco K., Cevallos W., Muñoz D., Acevedo A., Moser K.A., Marrs C.F. (2016). Antibiotic Resistance in Animal and Environmental Samples Associated with Small-Scale Poultry Farming in Northwestern Ecuador. Msphere.

[50] Lambert O., Piroux M., Puyo S., Thorin C., L’Hostis M., Wiest L., Buleté A., Delbac F., Pouliquen H. (2013). Widespread Occurrence of Chemical Residues in Beehive Matrices from Apiaries Located in Different Landscapes of Western France. PLoS ONE.

[51] Daghrir R., Drogui P. (2013). Tetracycline antibiotics in the environment: A review. Environ. Chem. Lett..

[52] Baggio A., Gallina A., Benetti C., Mutinelli F. (2009). Residues of antibacterial drugs in honey from the Italian market. Food Addit. Contam. Part B.

[53] Galarini R., Saluti G., Giusepponi D., Rossi R., Moretti S. (2015). Multiclass determination of 27 antibiotics in honey. Food Control..

[54] Er Demirhan B., Demirhan B. (2022). Detection of antibiotic residues in blossom honeys from different regions in Tur-key by LC-MS/MS method. Antibiotics.

[55] Kim D.-B., Song N.-E., Nam T.G., Jung Y.S., Yoo M. (2021). Investigation and human health risk assessment of multi-class veterinary antibiotics in honey from South Korea. J. Food Compos. Anal..

[56] Korkmaz S.D., Kuplulu O., Cil G.I., Akyuz E. (2017). Detection of sulfonamide and tetracycline antibiotic residues in Turkish pine honey. Int. J. Food Prop..

[57] Kumar A., Gill J., Bedi J., Chhuneja P. (2020). Residues of antibiotics in raw honeys from different apiaries of Northern India and evaluation of human health risks. Acta Aliment..

[58] Kumar A., Gill J.P.S., Bedi J.S., Chhuneja P.K., Kumar A. (2020). Determination of antibiotic residues in Indian honeys and assessment of potential risks to consumers. J. Apic. Res..

[59] Mahmoudi R., Norian R., Pajohi-Alamoti M. (2014). Antibiotic Residues in Iranian Honey by Elisa. Int. J. Food Prop..

[60] Wang Y., Dong X., Han M., Yang Z., Wang Y., Qian L., Huang M., Luo B., Wang H., Chen Y. (2022). Antibiotic residues in honey in the Chinese market and human health risk assessment. J. Hazard. Mater..

[61] Ahmed M.B.M., Taha A.A., Mehaya F.M.S. (2022). Method validation and risk assessment for sulfonamides and tetracyclines in bees’ honey from Egypt, Libya, and Saudi Arabia. Environ. Geochem. Health.

[62] Orso D., Floriano L., Ribeiro L.C., Bandeira N.M.G., Prestes O.D., Zanella R. (2015). Simultaneous Determination of Multiclass Pesticides and Antibiotics in Honey Samples Based on Ultra-High Performance Liquid Chromatography-Tandem Mass Spectrometry. Food Anal. Methods.

[63] Fahim H.M., Shaltout F., El Shatter M.A. (2019). Evaluate antibiotic residues in beef and effect of cooking and freezing on it. Benha Vet. Med. J..

[64] Saleh S.M.K., Mussaed A.M., Al-Hariri F.M. (2016). Determination of Tetracycline and Oxytetracycline Residues in Honey by High Performance Liquid Chromatography. J. Agric. Sci. Technol. B.

[65] Molino F., Lázaro R., Pérez C., Bayarri S., Corredera L., Herrera A. (2011). Effect of pasteurization and storage on tetracycline levels in honey. Apidologie.

[66] U.S. Residue Limits for Veterinary Drugs, Food Additives, and Unavoidable Contami-Nants in Meat, Poultry, and Egg Products. https://www.fsis.usda.gov/wps/wcm/connect/2fe2afb9-b935-4a74-83e0-587c41b2f784/2001_Residue_Limits_Veterinary_Drugs_App4.pdf?MOD=AJPERES.

[67] Baghani A., Mesdaghinia A., Rafieiyan M., Soltan Dallal M.M., Douraghi M. (2019). Tetracycline and ciprofloxacin multiresidues in beef and chicken meat samples using indirect com-petitive ELISA. J. Immunoass. Immunochem..

[68] Nisha A.R. (2008). Antibiotic Residues—A Global Health Hazard. Vet. World.

[69] Yorke J., Froc P. (2000). Quantitation of nine quinolones in chicken tissues by high-performance liquid chromatography with fluorescence detection. J. Chromatogr. A.

[70] Cerniglia C.E., Kotarski S. (1999). Evaluation of Veterinary Drug Residues in Food for Their Potential to Affect Human Intestinal Microflora. Regul. Toxicol. Pharmacol..

[71] Riley L.W., Raphael E., Faerstein E. (2013). Obesity in the United States–dysbiosis from exposure to low-dose antibiotics?. Front. Public Health.

[72] Ramatla T., Ngoma L., Adetunji M., Mwanza M. (2017). Evaluation of Antibiotic Residues in Raw Meat Using Different Analytical Methods. Antibiotics.

[73] Combs M.T., Boyd S., Ashraf-Khorassani M., Taylor L.T. (1997). Quantitative Recovery of Sulfonamides from Chicken Liver, Beef Liver, and Egg Yolk via Modified Supercritical Carbon Dioxide. J. Agric. Food Chem..

[74] O’Hara J.K., Woods T.A., Dutton N., Stavely N. (2021). COVID-19′s impact on farmers market sales in the Washington, DC, area. J. Agric. Appl. Econ..

